# Editorial: Substance use and the psychosis spectrum

**DOI:** 10.3389/fpsyt.2022.1004409

**Published:** 2022-08-18

**Authors:** Umut Kirli, Sinan Guloksuz, Hayriye Elbi

**Affiliations:** ^1^Institute on Drug Abuse, Toxicology and Pharmaceutical Science, Ege University, Izmir, Turkey; ^2^Department of Psychiatry, School of Medicine, Yale University, New Haven, CT, United States; ^3^Department of Psychiatry and Neuropsychology, School for Mental Health and Neuroscience, Faculty of Health, Medicine and Life Sciences, Maastricht University, Maastricht, Netherlands; ^4^Department of Psychiatry, Ege University, Izmir, Turkey

**Keywords:** substance and alcohol use, psychosis, extended psychosis phenotype, cannabis (marijuana), stimulant abuse or dependence, experience-sampling method (ESM), epidemiology-analytic (risk factors), neurobiological basis

## Introduction

Substance use rates have alarming trends of increase throughout the world (1). A recent multicentre study shows that the variation in substance use contributes to the variation in the incidence of psychotic disorders across different regions (2). Substance use is one of the most important modifiable risk factors for psychosis, which highlights the importance of research on the association between substance use and psychosis (3, 4). However, many questions on this association remain unanswered. In this article collection, we bring together some novel evidence from clinical, neurobiological as well as therapeutic perspectives.

## Papers in this research topic

Chang et al., present a cross-sectional study including 397 schizophrenia and related psychoses patients from a tertiary psychiatric hospital in Singapore. One in tenth of the patients reported problematic drug and/or alcohol use. This rate is relatively lower than the rates reported in western countries (1, 2). However, problematic drug/alcohol use is associated with greater mental distress and poor physical health in psychosis spectrum patients, in line with substantial evidence from different parts of the world (3–8).

To date, a considerable number of longitudinal studies have been conducted to elucidate the associations between substance use and psychosis (9–11). However, evidence on momentary dynamic associations between substance use and psychosis in daily life is scant. Weiss et al. present an interesting study using the experience-sampling methodology (ESM). In this study, youth with clinical high risk (CHR) or early psychosis (EP) provided data on substance use, psychotic symptoms, and negative affect six times a day *via* a smartphone application. Results showed that substance use was significantly associated with lagged negative affect. With relatively larger sample sizes and longer follow-up periods, ESM seems to be a promising tool to provide insight into moment-to-moment associations between substance use and psychosis.

Over the last two decades, clinicians struggle with the increased incidence of methamphetamine and ketamine induced psychoses across the world (12–14). However, our insight into this relatively “recent” crisis is limited. Luo et al. compared different features of psychotic symptoms between users of these drugs (n = 842). After at least two weeks of drug abstinence, psychotic symptoms were reported by three quarters of methamphetamine use disorder (MUD) patients and half of ketamine use disorder (KUD) patients. MUD patients were more likely to experience positive psychotic symptoms, as well as stereotyped thinking, difficulty in abstraction withdrawal, and poor rapport than KUD patients, whereas general symptoms, such as sleep and anxiety, were similar across the two groups.

This article collection includes novel studies on the neurobiological underpinnings of substance use-psychosis comorbidity. Johnstone et al. systematically reviewed the evidence on the clinical utility of neuromodulation techniques to treat comorbid substance use and psychosis spectrum disorder. Authors concluded that preliminary evidence supported the effectiveness of repetitive transcranial magnetic stimulation (rTMS) targeting the dorsolateral prefrontal cortex (DLPFC), a well-known brain region involved in top-down regulation, on reducing cannabis and tobacco use in patients with schizophrenia and schizoaffective disorder. However, some studies showed no significant effect of rTMS on reducing cannabis and tobacco use in this population, as also presented by Ward, Brady et al. in their mini review. An original fMRI study by Ward, Beermann et al. showed that nicotine use might normalize default mode network hyperconnectivity in patients with schizophrenia. Default mode network hyperconnectivity was previously associated with impaired attention. Considering the plausible unique pro-cognitive effect of nicotine use in patients with schizophrenia, this article suggested targeting the default mode network hyperconnectivity for smoking cessation in schizophrenia patients. Finally, Hau et al. presented a brief research report demonstrating a positive or borderline result of anti-neuronal autoantibodies in one-third of synthetic cannabinoid-induced psychosis patients. However, no significant association was found between PANSS scores and the presence of anti-neural antibodies in this study. These results require replication in larger samples.

One of the major challenges in psychiatry is the relatively low treatment adherence in patients with comorbid substance use disorders and psychosis spectrum disorder. Bouchard et al. present a comprehensive systematic review and meta-analysis of available evidence for dropout rates in psychosocial interventions for this population. Results showed that more than a quarter of these patients dropped out from psychosocial treatments with even higher rates among patients with stimulant use disorder. Long acting injectable antipsychotics (LAIs) might help cope with high drop-out rates. Coles et al. presented a systematic review of the literature investigating the use of LAIs in this population. Preliminary evidence suggests that LAIs might be safe, well tolerated, and mostly effective in the treatment of psychosis spectrum and substance use comorbidity. Furthermore, Gjerde et al. presented an original study demonstrating that cannabis use in patients with first treatment psychosis was not significantly associated with negative beliefs about antipsychotic medication. This preliminary result suggests that favorable treatment adherence may be achieved in comorbid psychosis spectrum and substance use disorder patients, if other reasons for non-adherence (e.g., lack of insight, side effects) are taken into account.

## Challenges and future directions

Substance use may be an excellent target for primary, indicated and selective prevention strategies for psychosis. However, there exist questions that await urgent answers to move the field forward. First, evidence on the causal link between substance use and different domains of psychosis (i.e., positive, negative, disorganization and affective) should be established in replication studies. Studies should also evaluate the possible influence of psychosis on substance use (i.e., reverse causality) by using genetically informed approaches, such as Mendelian randomisation analyses (15). Second, novel evidence for methods to intervene in substance use in individuals at high risk for psychotic disorders is needed. Third, insight on the link between excessive alcohol use and psychosis need to be clarified. Finally, urgent evidence on the role of different classes of substances (e.g., novel stimulants, gabapentinoids etc.) for inducing psychosis is needed. In summary, a clearer insight into the clinical and neurobiological basis of the link between substance use and psychosis warrant longitudinal studies with frequent follow-ups with shorter time gap between each visit and granular assessments that take into account genetic risk and environmental factors at both individual and neighborhood-based levels. We hope that this collection of interesting articles may draw scientific attention to this growing public health crisis (16).

## Author contributions

All authors listed have made a substantial, direct, and intellectual contribution to the work and approved it for publication.

## Conflict of interest

The authors declare that the research was conducted in the absence of any commercial or financial relationships that could be construed as a potential conflict of interest.

## Publisher's note

All claims expressed in this article are solely those of the authors and do not necessarily represent those of their affiliated organizations, or those of the publisher, the editors and the reviewers. Any product that may be evaluated in this article, or claim that may be made by its manufacturer, is not guaranteed or endorsed by the publisher.
